# Transactions of the Southern Surgical and Gynecological Association

**Published:** 1898-03

**Authors:** 


### TRANSACTIONS OF THE SOUTHERN SURGICAL AND GYNECOLOGICAL ASSOCIATION.

*Tenth Annual Meeting held in St. Louis, Mo., November, 1897.*

[Abstract.]

Dr. Howard A. Kelly, of Baltimore, read a paper entitled:

### SOURCES AND DIAGNOSIS OF PYURIA.

I BEGIN by stating that if I were asked what subject in the entire range of medicine and surgery I considered it most important to bring prominently before the profession at present, I

would probably reply, pyuria. The subject is important on account of the great number of undetermined cases under treatment and on account of the progressive nature of some forms of the disease, as well as on account of the facility with which the diagnosis can now be made with better means of investigation. Pyuria, of course, signifies merely the presence of pus in the urine ; this may be in large or in small quantities, and may proceed from any part of the urinary tract from the external urethral orifice up to the cortex of the kidneys.

The investigation of a pyuria is an analytical one, taking the symptom and trying to trace its origin. The best way to investigate a pyuria is to begin by making an examination of the urinary tract, following an anatomical order, proceeding from below upward. The history of the case, the microscopical and bacteriological and chemical examinations of the urine, must be carefully made, and all facts ascertainable by palpation must be elicited first. The direct investigation then proceeds in an orderly manner, beginning with the external urethral orifice, where Skene's glands may be distended with a drop or two of pus ; the urethra may be in a state of intense inflammation and even ulceration, affording sufficient pus to yield a decided sediment in the urine. Sometimes a suburethral abscess may pass unnoticed for many months, in spite of the fact that it contains from a teaspoonful to a tablespoonful of pus.

The vesical sources of pyuria are from a cystitis, including a trigonitis, or inflammation of the trigonum ; foreign bodies creating a cystitis ; ulcers associated with a cystitis or tubercular in their nature. These affections will all be readily recognised by making a cystoscopic examination of the patient in the knee-breast position through a simple cylindrical speculum. By this examination the cystitis will often be found to be well localised and in patches, which may be readily treated by direct topical applications. Upon removing a foreign body, the cause of a cystitis, the pyuria disappears. Ulcers seen through the cystoscope may be treated with strong solutions of nitrate of silver, curetted, or cauterised. There are also a number of extra sources of pyuria, and these arise most frequently from tubal and ovarian abscesses breaking into the bladder across the base of a broad ligament.

If the pyuria does not come from the lower urinary tract it must then come from one of the ureters or kidneys ; a telltale blush around a urethral orifice often marks the side from which the pus issues. I have found pyurias from the upper urinary tract proceeding from strictures of the ureter with a gonorrheal, tubercular, or other infection. The site of the stricture and the source of the pyuria may be readily located in these cases by passing a urethral catheter, a metal one for the low strictures or a flexible one for those above the pelvic brim.

Renal pyurias are, after all, the commonest of all, and I find that these are most frequently caused either by a calculus in the pelvis of the kidney or by a tubercular pyelitis or by a hydronephrosis which has become infected. Such pyurias are often the occasion of the presence of large amounts of pus in the urine, appearing either continuously or intermittently. By passing a renal catheter well up into the pelvis of the kidney the pus may be evacuated and the pelvis washed out. If the pus is too thick to flow through the catheter it may be thinned out by injecting a little fluid.

By making an orderly investigation of this sort the pus is traced to its origin, and the source of the disease discovered and treated and the cause eliminated, if possible, just as we would seek to investigate the source of the contamination of a body of water by taking a boat and traveling up the muddy stream until we had located the point at which it entered the main body, and until we found that above this point the water was free from contamination. The methods proposed are safe in careful, practised hands.

DISCUSSION.

Dr. Lewis S. McMurtry, of Louisville, Ky.: Dr. Kelly very appropriately remarked by way of introduction to his paper that the object of this society is educational. To promote such educational purpose it is essential that we should discuss one another's views freely, fairly and fearlessly, otherwise we will degenerate into a mutual admiration society, quite devoid of educational influence. Any criticism that I may offer on Dr. Kelly's paper will, I hope, be received in this spirit.

In the first place the subject presented by the essayist is not so novel as might at first appear. Diseases of the female bladder and urinary tract have been studied for years by Bozeman, Emmet, Skene, Simon and Pawlik. The published results of these investigations have illuminated the subject so that the diagnosis and treatment of diseases of the female bladder and urinary tract are now upon a scientific basis. Exploration of the bladder by endoscopy and of the ureters by catheterisation has been made practicable by Prof. Pawlik, and the method modified and popularised in this country by Dr. Kelly. As a diagnostic resource the method described by the essayist has certain definite value, but, in my judgment, its scope in practical therapy is almost *nil*. I have witnessed the use of the instruments by Dr. Kelly, and have personally applied the method in a number of cases of vesical and renal diseases, consequently I am discussing the subject from the standpoint of personal experience.

I would not be understood for a moment as saying that this method is useless and I beg that my remarks be not so construed. In the differential diagnosis between unilateral and bilateral disease of the kidney, in eliminating vesical involvement in cases of pyonephrosis and in some other conditions this method of exploration has a useful purpose and should be cultivated. It is altogether for diagnosis. I know no advantage it can offer in the treatment of renal disease.

It is the custom of surgeons to make an investigation of the urine of women as a routine part of examination preparatory to operation. In a very large proportion of the cases presenting for operation, pus will be found in the urine. This observation should not mislead the surgeon, since pyuria may be due to leucorrhea, gonorrhea or purely transient causes, disappearing when the operation has been performed. Unless there be quite a large quantity of pus, and in a specimen carefully drawn with the catheter, it is unnecessary to spend time and energy in search of a remote cause, when the most probable source of pus is apparent.

Moreover, exploration of the ureters is not under such conditions devoid of danger. Just as the area of gonorrheal infection may be extended from the vagina through the cervical canal and beyond by injudicious instrumentation, so may a vesical infection be carried along the ureteral mucous membrane to the pelvis of the kidney. Such indiscriminate and routine exploration of the upper genital tract in cases of pyuria as the essayist suggests is by no means a harmless procedure ; it may convey an infection along clean surfaces to the kidney. And again, ureteral catheterisation can avail nothing as a method of treatment. The constant secretion and passage of urine is as efficient for drainage here as any artificial washing can supply.

Dr. Kelly has cited a case of appendicular abscess opening into and discharging through the bladder as an instance illustrative of the value of this method in diagnosis and treatment. Surely such extensive disease would furnish in its clinical history and general physical signs sufficient indications for diagnosis and treatment, without searching through such a devious route. In another case the common clinical observation of suppurative salpingitis discharging into the bladder is mentioned in illustration. Such cases are not uncommon and I am unable to appreciate the necessity or value of vesical exploration, either for diagnosis or as an adjuvant to successful treatment of that form of pelvic abscess. Certainly one would be at a great disadvantage to exchange the established method of examination by touch for vesical exploration in such conditions, and I am again unable to appreciate how such exploration could materially add to the diagnostic signs of pelvic abscess with pyuria so readily detected by the ordinary methods now in vogue.

In the case of suppurative pyelitis just related by Dr. Henrotin, which was treated by both Dr. Kelly and himself, the ureter was catheterised and the pelvis of the kidney washed sixty-one times, when finally a nephrotomy was done for the cure of the patient. Is it an advantage to subject a patient to such prolonged and painful treatment of very doubtful efficacy when the more direct, more surgical and safe procedure of lumbar incision can be utilised? Certainly no better illustration of the inefficiency of ureteral catheterisation as a therapeutic resource could be given than the case related by Dr. Henrotin as treated by Dr. Kelly and himself.

In conclusion I venture to predict that the mature judgment which is born of experience will demonstrate that the range of usefulness of ureteral catheterisation is very limited. The enthusiasm attaching to a novel and ingenious procedure is prone to exaggerate its scope and value. The history of surgery is teeming with illustrative instances. While valuable in diagnosis, the successful treatment of pyuria involving the higher area must be conducted by other methods than ureteral catheterisation.

